# Transfer of invertebrates with hay during restoration operations of extensively managed grasslands in Switzerland

**DOI:** 10.1007/s10841-020-00282-8

**Published:** 2020-11-13

**Authors:** Ariane Stöckli, Daniel Slodowicz, Raphaël Arlettaz, Jean-Yves Humbert

**Affiliations:** Institute of Ecology and Evolution, University of Bern, Division of Conservation Biology, Baltzerstrasse 6, 3012 Bern, Switzerland

## Abstract

**Introduction:**

Hay transfer from a speciose donor meadow to a species-poor receiver grassland is an established method to restore species-rich grassland plant communities. However, it has rarely been investigated to which extent invertebrates can be transferred with hay during such operations, which was the aim of this study.

**Methods:**

Sampling was conducted in eight sites of the Swiss lowlands with one donor meadow and two receiver sites each. On the receiver sites, three to four white bed sheets of one square meter each were deployed on the ground to receive a standard quantity of fresh hay just transferred from the donor meadow. All living invertebrates were collected from these sheets with an aspirator and subsequently identified to order level.

**Results:**

On average (± SD), 9.2 ± 11.3 living invertebrates per square meter were transferred with the hay. Beetles were the most abundant species group, representing 46.9% of all transferred invertebrates, followed by true bugs (8.9%) and spiders (7.0%). More individuals were transferred when the donor meadow was mown with a hand motor bar mower than with a rotary disc mower. Similarly, more invertebrates were transferred when the hay was transported loosely with a forage wagon than compacted as bales.

**Discussion:**

While this study demonstrates that living invertebrates can be transferred with the hay, their subsequent survival and establishment remains to be explored.

**Implications for insect conservation:**

We recommend using a hand motor bar mower and a forage wagon for increasing the survival probability of invertebrates in hay transfer.

**Electronic supplementary material:**

The online version of this article (10.1007/s10841-020-00282-8) contains supplementary material, which is available to authorized users.

## Introduction

Several methods exist to actively restore or re-create grasslands. One commonly used method is the transfer of green, i.e. freshly mown hay from a species-rich donor grassland to a former arable land or species-poor receiver grassland, which was harrowed or ploughed beforehand (see Kiehl et al. 2010 for detailed description of the hay transfer method). The efficiency of the hay transfer method to increase plant or invertebrate diversity has been demonstrated in several studies (reviewed in Török et al. 2011 for plants, see Woodcock et al. 2010 for invertebrates). For example, Kiehl and Wagner (2006) found that 69–89% of the plant species from the donor grassland are transferred this way with the hay, with ca 66% being permanently established on the restored grassland after five years.

Invertebrates can also be trapped and transferred with the fresh hay in the same way. Indeed, Wagner (2004) demonstrated that *Metrioptera bicolor*, a grasshopper, can be directly transferred with this method. With a capture-mark-recapture approach, he established that 4.6% of the individuals capable to reproduce were transferred to a restored meadow. To the best of our knowledge, Wagner (2004) is the only study that investigated the potential of translocating invertebrates with hay. Furthermore, it remains unknown if other invertebrates than grasshoppers can be transferred this way.

The aim of this study was to identify and quantify, in terms of relative abundance, which invertebrates are effectively transferred with the hay from a donor to a receiver site. In effect, invertebrates have to survive several operations, including mowing, transportation and spreading of the hay (Humbert et al. 2010). Therefore, we hypothesized that the chances for a successful transfer of invertebrates are greater (1) when the donor meadow is mown with a lighter mowing machine (e.g. a bar mower instead of a rotary disc mower) and (2) when the hay is transported loosely and not compacted in bales.

## Materials and methods

### Experimental setup

**Fig. 1:**
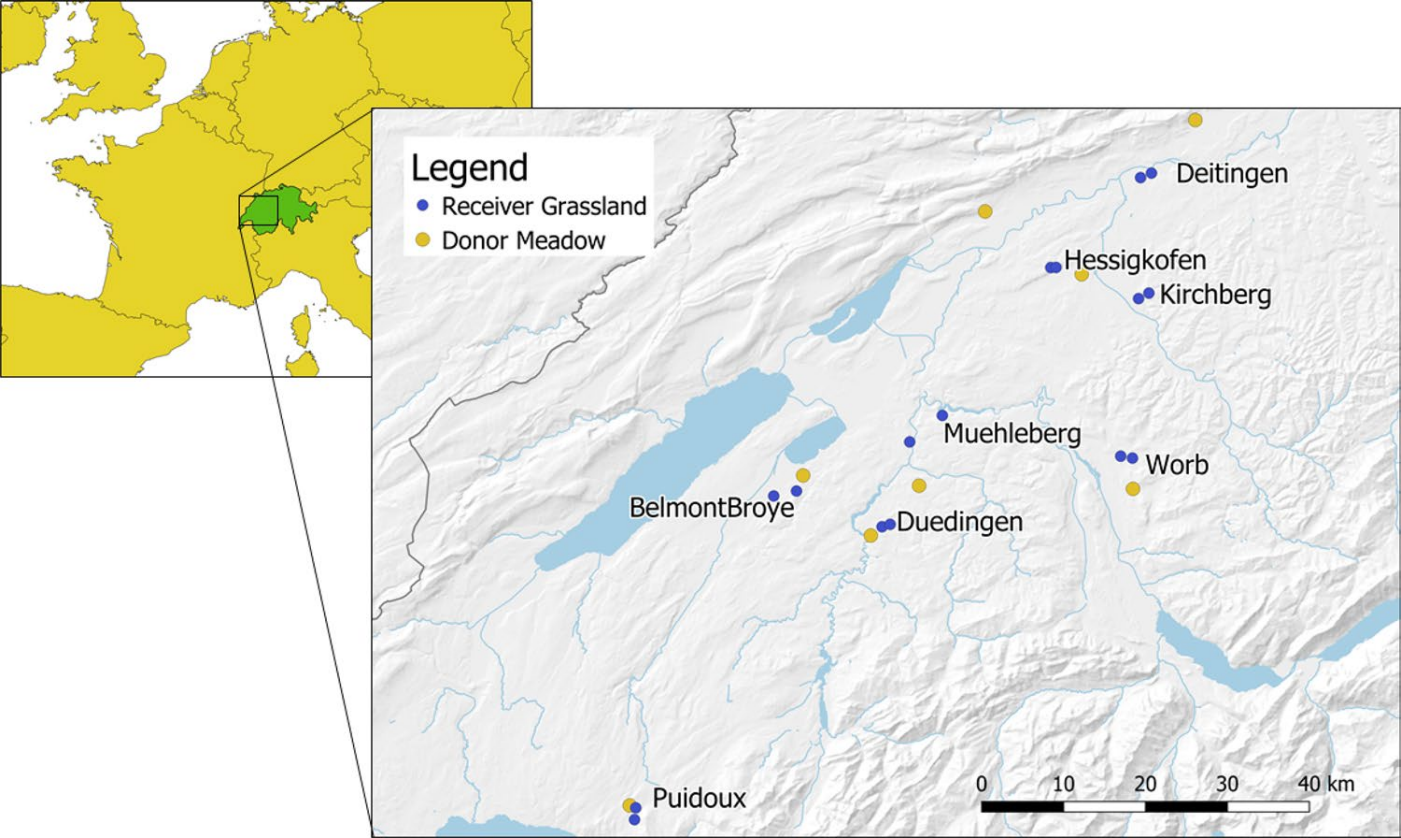
Study area in Switzerland. The donor meadows are represented with yellow dots, the receiver sites are represented with blue dots

**Fig. 2:**
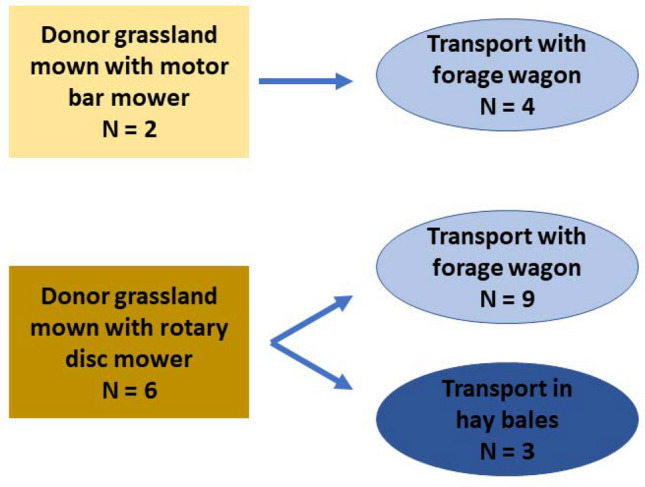
Overview of the mowing and hay transport techniques used in our experiment. Yellow squares represent donor meadows, blue circles represent receiver sites

The hay transfer and data collection were performed in June 2019 under warm and dry weather conditions. They took place in eight study sites located on the Swiss Plateau, an intensively-farmed lowland belt situated between the Alps and the Jura mountain ranges (elevation of study sites 423–712 m a.s.l., Fig. 1). Each site included one plant-speciose donor meadow (with 52–68 vascular plant species per meadow over the whole meadow and 26–47 vascular plant species within 2 × 4 m plots per meadow, meadow size 0.9–3.3 ha) and two receiver grasslands with a lower plant species richness (with 14–30 vascular plant species within 2 × 4 m plots per meadow, meadow size 0.2–0.9 ha). This resulted in a total of eight donor and 16 receiver meadows. Donor meadows were mesic hay meadows belonging to the Arrhenatherion elatioris community with a slight influence of the Mesobromion community. These meadows were managed extensively since at least 20 years, i.e. without fertiliser input and a first cut after June 15th. Receiver grasslands were also extensively managed since at least seven years. Prior to restoration, receiver meadows were either ploughed in March–April or harrowed just a few days before the transfer of the hay. To make the hay transfer possible within one day (i.e. mowing the donor meadow, transport the hay and spread it on the receiver site) and to avoid loss of seeds, the distance between the donor and receiver sites within a study site was not more than 10 km. In two sites, the donor meadows were mown with a hand motor bar mower, whereas at the other six sites a rotary disc mower was used. The transport of the hay was done for 13 meadows with a forage wagon and for three meadows as hay bales (Fig. 2). On each receiver site the hay was spread in a proportion of 1:1, i.e. 1 m^2^ of hay of the donor meadow was scattered on 1 m^2^ of the receiver site.

### Invertebrate sampling

**Fig. 3:**
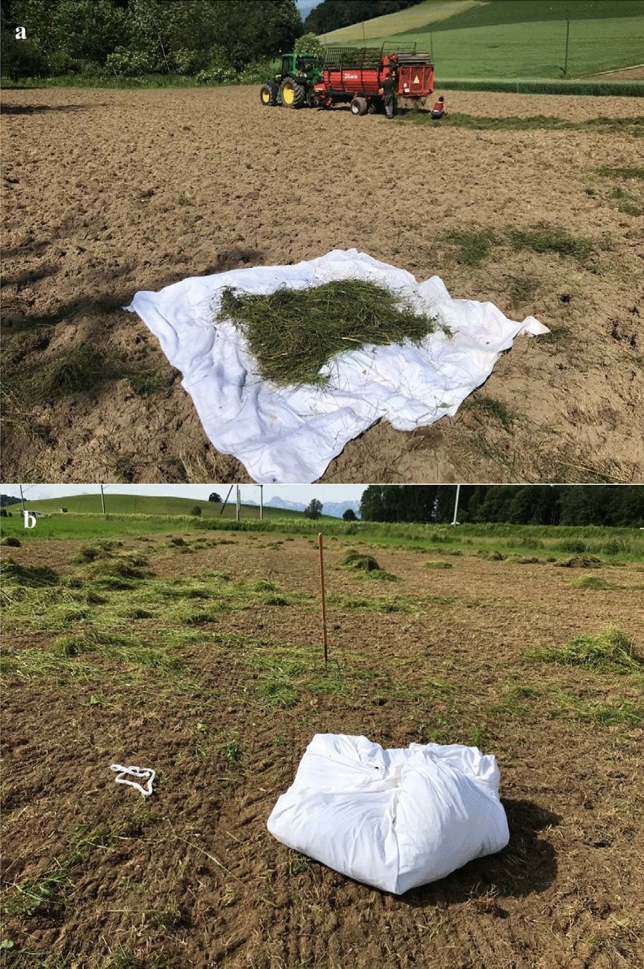
Field material: (**a**) a sampling linen bed sheet with the equivalent of 1 m^2^ of spread hay. In the background a forage wagon unloading the transferred hay onto the meadow; (**b**) sampling sheet closed to avoid living invertebrates to escape

Invertebrate sampling was carried out during the hay spreading operation. The hay was spread over three or four white 1 m^2^ linen bed sheets that were placed on the ground of any receiver meadow before the transfer. Each sheet received the freshly mown grass collected from 1 m^2^ of the donor meadow (Fig. 3a). Just after spreading the hay we closed the sheets to avoid invertebrates to escape (Fig. 3b). Next, we carefully opened the sheets and collected with an aspirator every living invertebrate that we could detect (i.e. > 1–2 mm). Ants were not collected because no survival was expected without their colony. Afterwards, the samples were stored in a freezer. In the lab we sorted and counted all sampled invertebrates to order level (in total 16 taxa).

### Data analyses

We analysed the quantity of transferred invertebrates with generalised linear mixed-effects models. Models were always run with the rounded average number of transferred invertebrates per meadow (two meadows per region) as response variable, whereas study site (spatial replicates) was set as a random effect. We first analysed the influence of the transfer technique by comparing the total number of invertebrates that were found after being transferred with a forage wagon (n = 13) or as hay bales (n = 3). Since the residuals were overdispersed, we corrected for it by adding an observer ID as a random effect. Secondly, the model was applied to assess the effect of the mowing machine, i.e. bar mower (n = 4) *vs* disc mower (n = 9). Due to the low sample size for hay bales (3 out of 16 receiver meadows) and the significant effect of the transport technique, only the data of forage wagon were used as an explanatory variable for the mowing machine analysis. All statistical analyses were performed with R version 3.5.1 (R Core Team 2018).

## Results

**Fig. 4:**
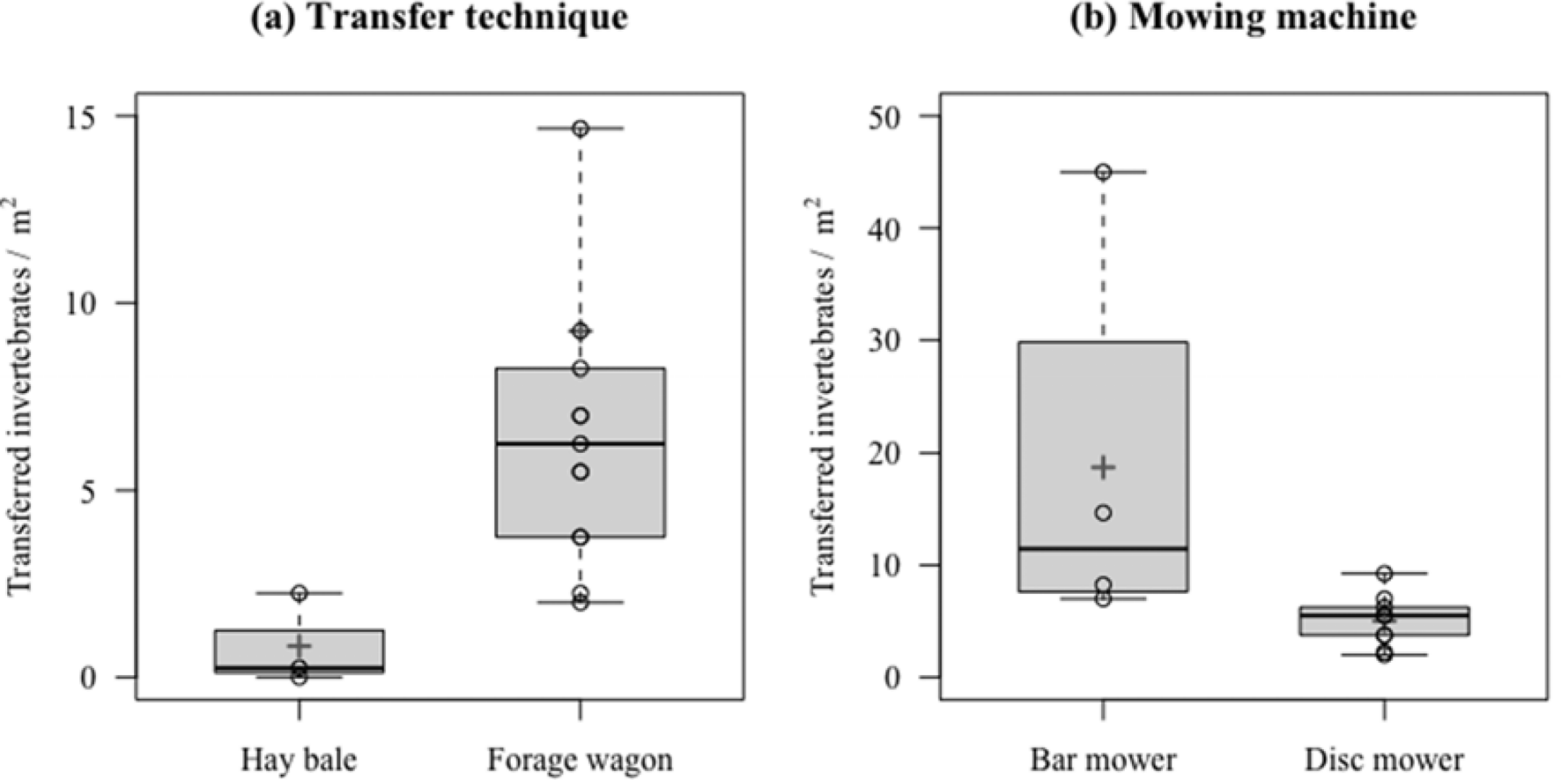
**a** Total number of transferred invertebrates per m^2^ with respect to the transportation technique: hay bale: 0.8 ± 1.2, n = 3 and forage wagon, (mean ± SD) 9.2 ± 11.3, n = 13. **b** Number of transferred invertebrates per m^2^ depending on the mowing machine: bar mower (mean ± SD) 18.7 ± 17.8, n = 4; and disc mower: 5.0 ± 2.3, n = 9. Means are represented as grey crosses

In total we sampled 429 invertebrates belonging to 16 taxa (Table 1, Appendix Fig. 5). The average number of transferred invertebrates per square meter ± SD (standard deviation) ranged between 9.2 ± 11.3 (n = 13) when the hay was transported from the donor to the receiver site with a forage wagon and 0.8 ± 1.2 (n = 3) with hay bales (estimate = 2.304, SE = 0.911, z = 2.529, *P* = 0.011; Fig. 4a). Beetles were the most abundant species group, representing 46.9% of all transferred invertebrates, followed by true bugs (8.9%) and spiders (7.0%). Although snails were the second most abundant group (9.3%), their fraction was lower than 1% when one site with super abundant snails was discarded. Larvae included all juvenile specimens, irrespective of whether they were attributable to a taxon or not (except for five sampled orthopterans that were all nymphs). Likewise, the type of mower had an influence on the number of transferred invertebrates: more invertebrates were transferred when the donor meadow was cut with a bar mower (n = 4) than with a disc mower (n = 9; estimate = 1.153, SE = 0.374, z = 3.08, *P* = 0.002; Fig. 4b).

**Table 1:** The proportion of transferred invertebrates on the receiver sites per taxa. In total 16 taxa were identified from 429 individuals

Taxa	Proportion (%)
Beetles	46.9
Snails	9.5
Larvae	9.3
True bugs	8.9
Spiders	7.0
Sternorrhyncha	5.1
Flies	4.4
Earwigs	3.3
Auchenorrhyncha	1.9
Orthopterans	1.2
Hymenopterans	1.2
Isopods	0.5
Caddisflies	0.2
Net-winged insects	0.2
Lepidopterans	0.2
Springtails	0.2

Juveniles of each taxa were pooled in “larvae”, expect for orthopterans where only nymphs were found and are represented as an own group

## Discussion

This study shows that a variety of living invertebrate taxa can be successfully transferred from a donor to a receiver meadow with the hay transfer method. It further suggests that when a forage wagon is used for transporting the freshly cut hay, 9.2 invertebrates per m^2^, on average, were transferred. Extrapolated to one hectare this figure sums up to 92,000 transferred individuals. Given that the detectability of smaller invertebrates is generally low, this figure should be considered as conservative.

We do not know the original invertebrate densities in the donor meadows for 2019, but true bugs and spiders were sampled in these same eight donor meadows in 2018 using suction sampling (as in Buri et al., 2016). Looking only at the donor meadows for which a forage wagon had been used, we sampled, in 2018, on average, 21 adult true bugs and 49 adult spiders per m^2^ (unpublished data). Therefore, assuming similar population densities in 2018 and 2019, we can estimate an average transfer rate of 2.5% (median 1%, range 0–10%) for true bugs and 2.3% (median 0.7%, range 0–14%) for spiders. Regarding beetles, we have no previous quantitative estimates of densities as they were sampled with pitfall traps, which cannot be related to a reference sampling area.

Ten times more living invertebrates were transferred when a forage wagon was used compared to bales, although sample size for the latter method was small. This was expected as baler machines compact the hay, including animals trapped in it, much harder than forage wagons. Although we could not find studies on the effect of baling on the survival of invertebrates, we expect it to be much lower due to the impact of compaction. Similarly, fewer invertebrates were transferred when the donor meadows were cut with a rotary disc mower than with a hand motor bar mower. This is probably due to the higher mortality induced by rotary mowers, which are powered by tractors, than by hand motor bar mowers that have light engines (Humbert et al. 2010). Although the type of mowing machine had a significant influence on the number of transferred invertebrates, it has to be taken into account that the overall sample size was also disproportionally smaller for the bar mower.

Although this study demonstrates that many living invertebrates are transported via the hay transfer method typically applied in active grassland restoration operations, it measured neither the survival nor the establishment success of the translocated invertebrates. To constitute a new viable population, a minimum number of individuals should be transferred (Shaffer 1981). Gardiner (2010) showed that translocating 40 adult individuals (sex ratio of 1:1) of the orthopteran *Myrmeleotettix maculatus* led to reproduction the following year. Berggren (2001) obtained a minimum population size of 32 individuals for efficiently translocating the orthopteran *Metrioptera roeseli* to previously uninhabited meadows. However, at the time of hay transfer, the vegetation is very scarce or not present, which might represent a serious impediment to invertebrate installation, notably of herbivorous species. Especially for less mobile species it is more difficult to move to more densely vegetated field margins or adjacent meadows (Thorbek and Bilde 2004). To circumvent the issue of a non-vegetated receiver site, a second hay transfer after the restoration of the plant community may be foreseen as an option to further increase invertebrate diversity and abundance (Kiehl and Wagner 2006). Another option would be to set aside an unploughed vegetated meadow patch or strip on the receiver site, which can serve as refuge during the vegetation free period (Humbert et al. 2012). In addition, Woodcock et al. (2010) found that invertebrates can recolonize restored meadows after hay transfer, once that a more diverse plant community is established. The recolonization rate of invertebrates, however, depends on the landscape and connectivity to other source populations.

In light of our results, we recommend to mow the donor meadow with a hand motor bar mower and transport the fresh hay with a forage wagon. This will maximize the total number of transferred living invertebrates and thus increase the probability of establishment. Given that hand motor bar mowers are smaller and therefore more time consuming in mowing grass, this approach is only feasible on small meadows.

### Electronic supplementary material

Below is the link to the electronic supplementary material.

Supplementary file1 (XLSX 17 KB)

## Figures and Tables

**Fig. 5:**
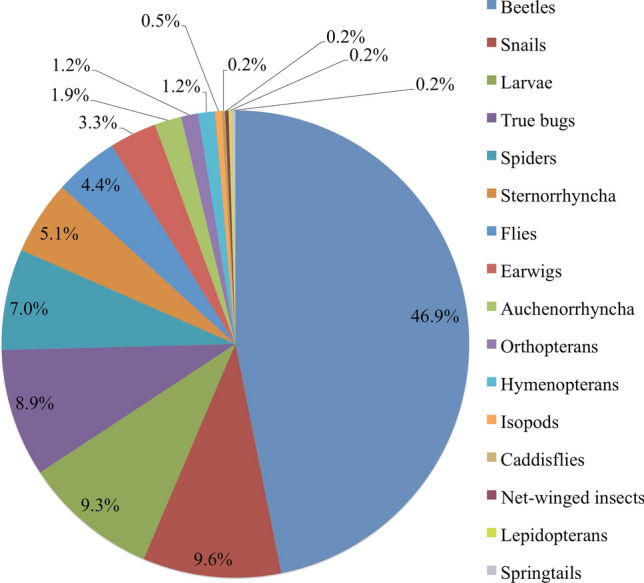
Overview of transferred invertebrates on the receiver meadows. In total 16 taxonomic groups were identified. Juveniles of each group were pooled in the category “larvae”, except for orthopterans where only nymphs were found and are thus represented as an own group

## Appendix

See Fig. 5

## References

[CR1] Berggren Å (2001). Colonization success in roesel’s bush-cricket Metrioptera roeseli: The effects of propagule size. Ecology.

[CR2] Buri P, Humbert JY, Stańska M (2016). Delayed mowing promotes planthoppers, leafhoppers and spiders in extensively managed meadows. Insect Conserv Divers.

[CR3] Gardiner T (2010). Successful translocation of the locally rare mottled grasshopper Myrmeleotettix maculatus to Jaywick flood defences in Essex, England. Conserv Evid.

[CR4] Humbert JY, Ghazoul J, Richner N, Walter T (2010). Hay harvesting causes high orthopteran mortality. Agric Ecosyst Environ.

[CR5] Humbert JY, Ghazoul J, Richner N, Walter T (2012). Uncut grass refuges mitigate the impact of mechanical meadow harvesting on orthopterans. Biol Conserv.

[CR6] Kiehl K, Wagner C (2006). Effect of hay transfer on long-term establishment of vegetation and grasshoppers on former arable fields. Restor Ecol.

[CR7] Kiehl K, Kirmer A, Donath TW (2010). Species introduction in restoration projects - evaluation of different techniques for the establishment of semi-natural grasslands in Central and Northwestern Europe. Basic Appl Ecol.

[CR8] R Core Team (2018) R: a language and environment for statistical computing. R Foundation for Statistical Computing, Vienna

[CR9] Shaffer ML (1981). Minimum population sizes for species conservation. Bioscience.

[CR10] Thorbek P, Bilde T (2004). Reduced numbers of generalist arthropod predators after crop management. J Appl Ecol.

[CR11] Török P, Vida E, Deák B (2011). Grassland restoration on former croplands in Europe: an assessment of applicability of techniques and costs. Biodivers Conserv.

[CR12] Wagner C (2004) Passive dispersal of Metrioptera bicolor (Phillipi 1830) (Orthopteroidea: Ensifera: Tettigoniidae) by transfer of hay. J Insect Conserv 8:287–296. doi:10.1007/s10841-004-0404-x

[CR13] Woodcock BA, Vogiatzakis IN, Westbury DB (2010). The role of management and landscape context in the restoration of grassland phytophagous beetles. J Appl Ecol.

